# Rules and Exceptions: The Role of Chromosomal ParB in DNA Segregation and Other Cellular Processes

**DOI:** 10.3390/microorganisms8010105

**Published:** 2020-01-11

**Authors:** Adam Kawalek, Pawel Wawrzyniak, Aneta Agnieszka Bartosik, Grazyna Jagura-Burdzy

**Affiliations:** Department of Microbial Biochemistry, Institute of Biochemistry and Biophysics, Polish Academy of Sciences, Pawińskiego 5a, 02-106 Warsaw, Poland; a.kawalek@ibb.waw.pl (A.K.); wawrzyniakp@ibb.waw.pl (P.W.); anetab2@ibb.waw.pl (A.A.B.)

**Keywords:** ParB, segrosome, chromosome segregation, cell division, gene expression regulation, partitioning proteins

## Abstract

The segregation of newly replicated chromosomes in bacterial cells is a highly coordinated spatiotemporal process. In the majority of bacterial species, a tripartite ParAB-*parS* system, composed of an ATPase (ParA), a DNA-binding protein (ParB), and its target(s) *parS* sequence(s), facilitates the initial steps of chromosome partitioning. ParB nucleates around *parS*(s) located in the vicinity of newly replicated *oriC*s to form large nucleoprotein complexes, which are subsequently relocated by ParA to distal cellular compartments. In this review, we describe the role of ParB in various processes within bacterial cells, pointing out interspecies differences. We outline recent progress in understanding the ParB nucleoprotein complex formation and its role in DNA segregation, including ori positioning and anchoring, DNA condensation, and loading of the structural maintenance of chromosome (SMC) proteins. The auxiliary roles of ParBs in the control of chromosome replication initiation and cell division, as well as the regulation of gene expression, are discussed. Moreover, we catalog ParB interacting proteins. Overall, this work highlights how different bacterial species adapt the DNA partitioning ParAB-*parS* system to meet their specific requirements.

## 1. Introduction

Bacterial genomes consist of circular or linear and single or multiple chromosomes as well as extra-chromosomal elements like plasmids. They are highly compact spatially and temporally organized entities [1,2,3,4]. Chromatin organization is linked to processes such as DNA replication and chromosome segregation and transcription [1,5,6,7,8,9]. In dividing cells, a bi-directional, semi-conservative replication initiated from *oriC* proceeds simultaneously with the segregation of compacted daughter nucleoids. Specific factors, such as DNA supercoiling, and proteins, such as nucleoid associated proteins (NAPs), determine, maintain, and modify the spatial organization of bacterial nucleoids during the entire cell cycle, from the initiation of replication to the end of the division cycle (reviewed in [2,10,11,12]).

Bacterial genomes are divided into macrodomains 0.5–1.5 Mbp in size, with the ori domain (up to 20% of the genome around the origin of replication *oriC*) and ter domain (part of the chromosome around the terminus of replication) playing pivotal roles [13,14,15,16,17,18]. A chromosome interaction map constructed for *Caulobacter crescentus* revealed that its genome is additionally split into 23 self-interacting regions of 30–400 kbp, designated chromosome interacting domains (CID) [9]. A similar number of CIDs ranging in size from 50 to 300 kbp were identified in the *Bacillus subtilis* genome [16]. The boundaries of CIDs frequently co-localize with highly transcribed genes [9,19,20,21,22]. The role of NAPs in chromosomal long- and short-range interactions [4,23,24] and among them, the structural maintenance of chromosome proteins (SMCs and MukBs) [25,26,27,28], has been widely documented. In *Mycoplasma pneumoniae*, 10–15 kb microdomains with similar gene expression patterns were identified [29].

Chromosomes in most rod-like bacteria, including *B*. *subtilis, C. crescentus, Pseudomonas aeruginosa, Vibrio cholerae*, and *Myxococcus xanthus*, adopt longitudinal organization. In this arrangement, the two replication arms align along the long axis of the cell, whereas the ori and ter domains locate at opposite cell ends [9,16,28,30,31]. The left and right arms are thought to wrap around each other, leading to a juxtaposition of the corresponding fragments of opposite chromosome arms [32,33,34]. The *Escherichia coli* chromosome exemplifies a transversal configuration, with the ori and ter localized in the middle of the cell and the two replication arms occupying distinct cell halves [17,35,36]. The dynamic and species-specific movements of particular chromosomal regions suggest that different factors may be involved in their organization and spatiotemporal segregation.

In this review, we summarize our current understanding of chromosomal ParAB-*parS* partition systems, which are involved in ori positioning in many species. We focus on the diverse roles of the ParB component and present recent advances in the ParB nucleoprotein complex formation, its involvement in DNA segregation, and other more specialized functions. Moreover, we catalog ParB interacting proteins from various species, which indicates that the biological roles of ParAB-*parS* systems may extend far beyond the chromosome segregation process.

## 2. ParAB-*parS*-Driven DNA Segregation—From Plasmids to Chromosomes

The accurate distribution of genetic material in bacteria was initially studied for low-copy-number plasmids, which secure their maintenance in the bacterial population by active DNA partition processes [37,38,39,40]. The vast majority of such plasmids uses three component Par systems built from an operon encoding an NTPase (A component, “motor protein”) together with a DNA-binding protein (B component), as well as *parS*, a centromere-like DNA sequence [41,42,43]. NTPases belong to Walker-type ATPases (partition system of Class I, ParA superfamily) [44,45], actin-like ATPases (Class II) [46], or tubulin-like GTPases (Class III) [47]. The B components use either helix-turn-helix (HTH) (ParB family) [48,49,50,51], ribbon-helix-helix (RHH) [52,53,54], or winged HTH motifs [55] for DNA binding to *parS*: specific inverted or direct sequence repeats occurring in a single or multiple copies in the plasmid genomes. Despite the structural variability of ParAB-*parS* elements between plasmids, the main steps of active plasmid partition are conserved. The B component specifically binds to *parS* site(s) and forms a nucleoprotein complex called a segrosome. The segrosome attracts NTPase, which, in turn, actively separates segrosome pairs by moving individual segrosomes towards the poles of dividing cells [56]. This cellular localization assures that each progeny cell will obtain at least one copy of the plasmid DNA (reviewed in [15,57]).

Studies on plasmid partition received special attention when the sequencing of bacterial genomes revealed *oriC* proximal operons encoding Class Ia plasmidic orthologs, ParAs, Walker-type ATPases, and ParBs, large proteins with a central HTH motif [41,58,59,60]. The third element, the variable plasmid-specific sequence(s), are replaced by a highly conserved palindromic *parS*s, mainly TGTTNCACGTGAAACA, present in varying numbers in the vast majority of single chromosomes, as well as the primary chromosomes of multipartite bacterial genomes (Table 1). To date, *par* genes have not been found in the chromosomes of the two families of γ-proteobacteria, *Enterobacteriaceae* (e.g., *E. coli*) and *Pasteurellaceae* (e.g., *Haemophilus influenzae*), or in one family of Mollicute, *Mycoplasmataceae* (e.g., *Mycoplasma* sp). Moreover, a few species seem to miss particular *par* elements (e.g., *Streptococcus pneumoniae* lacks *parA*) [41,59].

The homology between the chromosomal and low-copy-number plasmid counterparts of ParA and ParB families implicates their participation in DNA segregation. Indeed, chromosomal *par* operons accompanied by a single *parS* site from the same genome were shown to stabilize otherwise unstable plasmids and Par proteins were able to correctly position the plasmids even within heterologous host cells [61,62,63,64]. Undoubtedly the chromosome segregation process faces more spatiotemporal challenges than the partition of small plasmid genomes. 

**Figure 1:**
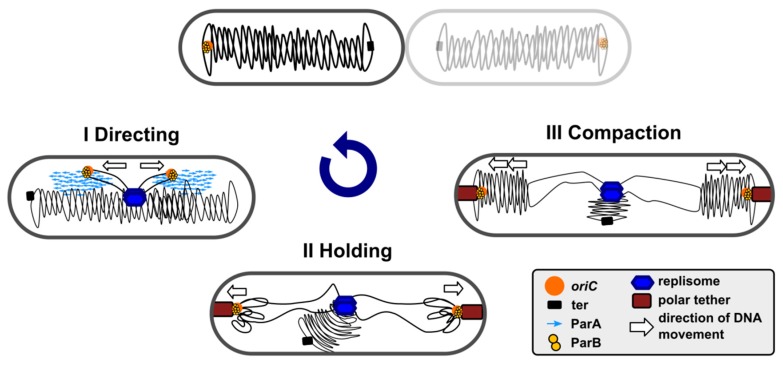
Involvement of partition ParB protein in ori domain re-locations, structuring and positioning during bacterial cell cycle.simplified scheme for *P*. *aeruginosa*-like longitudinal chromosome rearrangements is presented, in which the replisome is located in the cell centre, and the ParB-bound ori domains are anchored close to the cell poles before division [30].

The segregation of simultaneously replicating chromosomes proceeds in a few stages. The newly duplicated ori domains are re-folded and pushed away, directed, and held at certain cellular positions. The bulk of chromosome arms follows them, and finally, the ter domains become physically separated [2,65,66,67]. Studies on the role of ParAB-*parS* systems have revealed their engagement in ori domain compaction, directional movements, and specific positioning in the cell until cell division (Figure 1), in a species-specific manner. These systems are indispensable in some species, such as *C. crescentus* or *M. xanthus*, and are an accessory in the majority of other species (Table 1). 

Sequence analysis revealed that the vast majority of the ParA homologs encoded in the chromosomes of Gram-negative and Gram-positive bacteria, as well as Archaea, cluster in a subgroup, distinct from the groups of plasmidic Class Ia ParAs [41]. Chromosomal ParAs lack the N-terminal DNA binding domain, present in plasmidic orthologs and required for autoregulation of the cognate *par* operon [99,100].

Chromosomal ParAs, like their plasmidic counterparts of Class I, are Walker-type ATPases capable of non-specific DNA binding [101,102] and interactions with cognate ParBs [64,103,104,105]. After ATP binding, chromosomal ParAs associate non-specifically with the nucleoid. ATP hydrolysis stimulated by interactions with ParB bound to *parS*s triggers dynamic re-locations of the *oriC* domains. Several models have been offered to explain the molecular mechanisms used by the ParAs of Class I to move the ParB-*parS* complex towards the poles, including ”pulling” [42,103,106,107,108], “diffusion-ratchet”, and “DNA-relay” [109,110,111] and these mechanisms are still being discussed (for a review, see [15,112,113]).

**Figure 2:**
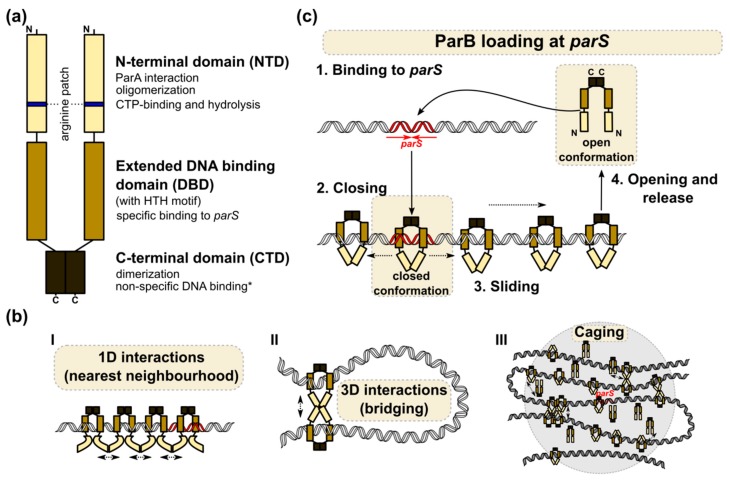
ParB complex assembly at *parS*. (**a**) Schematic representation of chromosomally encoded ParB protein (dimer) with the indicated functions of individual domains. *- only confirmed for *B*. *subtilis* Spo0J. (**b**) Models of the ParB–ParB interactions involved in formation of the ParB nucleoprotein complexes around *parS*. (I) Adjacent ParB dimers may interact with each other to form 1D filaments around *parS*. (II) Interactions between ParB dimers associated with distal DNA fragments may lead to DNA bridging and looping. (III) ParB self-interactions provide a scaffold (cage), attracting and trapping additional ParB molecules. (**c**) A model illustrating ParB loading at *parS* and sliding [122]. Free CTP-ParB exists as a dimer in an open conformation. Binding to *parS* induces conformational changes involving the N-terminal ParB domains and the formation of “closed” ring-shaped molecules. Steric hindrance between HTH motifs interacting with *parS* in such a closed conformation may prompt the release of ParB rings from *parS* via their sliding on adjacent DNA and the loading of new ParB dimers at *parS*. Finally, switching from a closed to open conformation by an unknown mechanism (possibly involving CTP hydrolysis) may lead to ParB’s dissociation from the DNA. The *parS* sites are indicated in red.

Eubacterial chromosomal ParBs (but not archaeal), and plasmid encoded Class Ia partition proteins [41], belong to the ParB family. Chromosomal ParBs cluster together and show a much higher conservation in their clade than more divergent plasmidic ParBs. Despite their apparent diversity, all Class Ia ParBs share a similar domain and functional organization (Figure 2a). The central DNA binding domain (DBD) with an extended HTH motif is connected by flexible linkers, with the N-terminal oligomerization and ParA binding domain (NTD) containing the highly conserved arginine patch, GERRxRA [114,115,116,117] (reviewed in [118]), and the C-terminal dimerization domain (CTD) encompassing a leucine zipper [64,116,119]. Additionally, in some systems, CTD may be involved in nonspecific DNA interactions (e.g., *B*. *subtilis* ParB homolog, designated Spo0J) [119,120,121].

## 3. The Structure of the ParB–*parS* Complex

Similarly to their plasmidic orthologs [123,124,125,126], chromosomal ParBs have a very unusual feature: after binding to *parS* as dimers, they spread on adjacent DNA [64,68]. It was shown that ParBs defective in spreading were impaired in partition [68,114,127].

ParB nucleation around the *parS* sequence correlates with the formation of ParB foci in fluorescence microscopy analyses [76,92,97,117,127,128,129,130,131] or the presence of wide peaks (up to 50 kbp), encompassing *parS* in chromatin immunoprecipitation analyses (ChIP-seq or ChIP-microarray), indicating the incorporation of *parS* proximal DNA in the ParB complex [68,71,72,79,80,89,90,129,132]. 

A combination of biochemical, structural, and computational approaches can shed light on the possible architecture of the ParB–*parS* nucleoprotein complex (reviewed in [118]). Nevertheless, no common assembly mechanism has been proposed, thus suggesting dynamic and heterogeneous interactions between ParB molecules within the complex (Figure 2b) [116,122,133,134]. It is widely acknowledged that ParB loading on *parS* is a prerequisite for the conformational changes that prime ParB for nucleation [135,136,137]. The ability of ParBs to build large nucleoprotein complexes may be a result of lateral ParB interactions (1D) and bridging interactions (3D) between ParB molecules located at distant DNA segments [117,134], clustering or building a ParB cage around *parS* by weak but dynamic interactions between protein dimers and DNA [129,138,139]. The ParB interactions around *parS* result in significant DNA compaction via loop formation. Interestingly, non-specific DNA binding also seems to be an important factor in DNA bridging and condensation, at least in some systems [120,121]. All models postulate that multiple ParB–ParB interaction interfaces must be involved in the assembly of higher-order complexes. Structural studies have indeed demonstrated the flexibility of ParB molecules, the ability to bridge different *parS* sequences, as well as various cross-monomer interactions [115,116,122,133]. Notably, the majority of mutational analyses show that the conserved arginine patch residues are required for ParB spreading and DNA partition, thereby indicating the crucial role of this motif in ParB functions [68,114,127]. 

Mechanistic insight into the loading of *B. subtilis* Spo0J on *parS* and the role of the conserved arginine patch was recently provided [122]. Spo0J was shown to hydrolyze cytidine triphosphate (CTP) with the catalytic center encompassing the arginine patch **G**ER**R**FR**A** (nucleotide binding residues in bold). In the presence of *parS*, an open form of the CTP-bound Spo0J dimer favors cooperative CTP binding and closes into a ring-shaped ParB clamp on *parS* (Figure 2c). The steric hindrance between *parS* bound HTH motifs was suggested to promote the detachment of the ParB dimers in a closed conformation from *parS*, as well as sliding away (spreading). CTP hydrolysis is not required for loading but might instead assist ParB recycling and control ParB spreading, as shown *in vitro* for *C. crescentus* ParB [140]. Spreading may also be restricted by road blocks formed by NAPs [122,140]. The role of CTP binding and hydrolysis in ParB-driven partition complex formation was also shown for *M. xanthus* ParB [141]. Interestingly, in a recent study, Jalal and co-workers demonstrated that ParBs from various bacterial species show variation in their intrinsic capabilities for spreading and that the determinant of this variability maps to the N-terminal domain (NTD) [133]. The study used ChIP-seq to analyze ParB spreading in a heterologous host, so the results may not recapitulate all the determinants affecting the extent of spreading in its original host (like DNA supercoiling, involvement of other proteins). Nevertheless, this demonstrates that the NTD domain might evolve to regulate ParB’s association with DNA. 

## 4. ParB Binding to Half-*parS*: A Novel Aspect of ParB–DNA Interactions?

A recent ChIP-seq analysis of ParB binding in *P. aeruginosa* added a new dimension to ParB–chromosome interactions [90]. In addition to the ParB-enrichment at *parS*s [64,91] hundreds of additional sites containing a half-*parS* motif (mainly GTTCCAC or GTTTCAC) were also shown to be occupied by *P*. *aeruginosa* ParB [90]. While ParB binding to four *parS*s proximal to *oriC* resulted in a 50 kbp peak, the width of the ParB peaks around the half-*parS* sites did not exceed 0.6 kbp, even under an abundance of ParB, suggesting a distinct mode of interactions.

**Figure 3:**
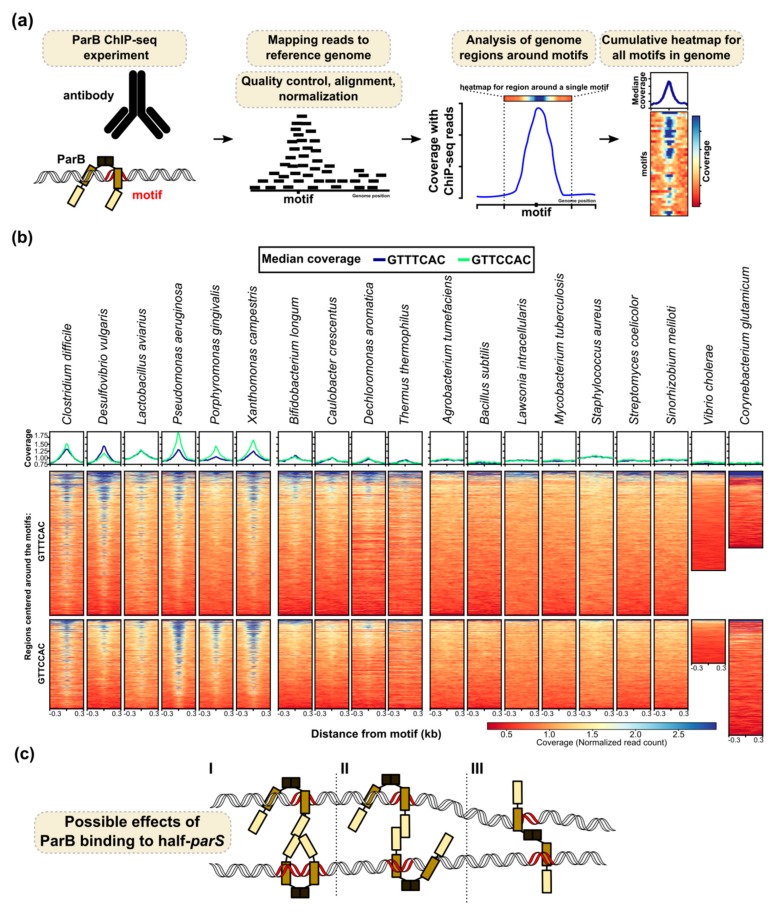
Interactions of ParBs with half-*parS* sites. (**a**) Outline of the analysis of ParB binding to half-*parS* using the available ParB ChIP-seq data. (**b**) The binding of ParB proteins from various bacterial species to half-*parS*s assessed by the enrichment of half-*parS* (GTTCCAC and GTTTCAC) containing genomic DNA fragments in the ChIP samples. Heatmaps represent the read coverage for the ParB ChIP samples, calculated for each nucleotide of a ±300 bp region around all the indicated motifs in the corresponding reference genomes. Plots represent median coverage. A central increase of the coverage indicates enrichment of the DNA containing motif during chromatin immunoprecipitation for the corresponding ParB protein; hence, ParB binds to these sequences. The ChIP-seq data for 17 ParBs from different species produced in *E*. *coli* [Gene Expression Omnibus GSE129285 [142]], *V*. *cholerae* ParB1 [GSM3161909, GSM3161911 [129]], and *C*. *glutamicum* ParB [SRX5581454, SRX5581458, SRX5581460 [72]] were included in the analysis. Raw data were downloaded from the sequence read archive (SRA) and quality-controlled using fastp [143]. Reads were mapped to the reference genomes of *E*. *coli* K-12 substr. MG1655 (U00096.3), *V. cholerae* O1 biovar El Tor str. N16961 (only chrI, NC_002505.1), and *C. glutamicum* ATCC 13032 (BX927147), respectively, using Bowtie [144] with the --sensitive-local option. Samtools was used to exclude duplicate reads and sort the .bam files [145]. Coverage (.bigwig) files were generated with bamCoverage [146], using the --normalizeUsing RPGC option, without binning and smoothing. Half-*parS* motifs (GTTCCAC and GTTTCAC) were identified in the corresponding genomes using fuzznuc (Emboss 6.6.0). Heatmap displaying coverage with reads in the ParB ChIP data around the identified motifs were generated using plotHeatmap from deepTools [146]. Each line in the heatmap represents the normalized read counts for each nucleotide of a ±300 bp region around one motif, sorted in the descending order of the mean coverage value and colored according to scale. The median coverage score for the two sets of motifs is presented on plots above the heatmap. For *V*. *cholerae* and *C*. *glutamicum*, the data from the biological replicates were averaged. (**c**) Hypothetical model engaging the half-*parS* sites in DNA structuring. I—The ParB complex loaded at *parS* interacts with an “open” dimeric ParB bound to a half-*parS* site. II—Interactions between two ParB dimers bound to separate half-*parS*s. III—Both monomers in a ParB dimer interact with separate half-*parS*s. All scenarios result in the formation of DNA bridges. In this model, we assume that the binding of ParB to half-*parS* involves the HTH in the central domain.

Interestingly, our analysis of available ChIP-seq data (including the 17 chromosomal ParBs from various species produced in *E. coli* [142] and tested for DNA binding in this heterologous host, as well as two ParBs from *V. cholerae* and *Corynebacterium glutamicum* tested in their native hosts) showed that binding to *parS* half-sites GTTCCAC and GTTTCAC is not a unique feature of *P. aeruginosa* ParB (Figure 3a,b). ParB of *P. aeruginosa* and five other ParBs clearly bind to these heptanucleotides even in a heterologous host. Four ParBs show slightly weaker binding, whereas among the remaining nine including *B. subtilis*, *S. coelicolor*, *V. cholerae*, and *C. glutamicum*, no binding to the selected motifs can be observed. It is feasible that the presence of hundreds of specific ParB–DNA binding sites in the genome enables an additional role of this protein in the modulation of chromosome topology, possibly through the interactions of ParB bound to the half-*parS* with the ParB complex assembled at *parS*s, or through bridging distant DNA segments (Figure 3c). These interactions may play a role in local or global DNA condensation in a species-dependent manner.

## 5. The Role of ParBs in DNA Topology 

The architecture of the origin domain plays an important role in the regulation of replication initiation, global chromosome organization, and DNA segregation [5,16]. The conserved feature of the chromosomal ParAB-*parS* systems is the localization of the *par* operons and the vast majority of *parS* sites within the so-called ori domain, defined as 20% of the chromosome around *oriC* [58,59], suggesting a functional relation. Among all studied bacteria with complete ParAB-*parS* systems, ParB homologs perfectly mark and position the *oriC* regions within the cell, and their absence impairs proper *oriC* localization during the cell cycle [68,71,72,76,82,88,92]. The majority of bacterial species utilizing ParAB-*parS* for DNA segregation contain more than one *parS* sequence close to *oriC* (Table 1). Nevertheless, a single *parS* site in the vicinity of *oriC* is enough to secure the proper positioning of *oriC* in the cell and segregation of the duplicated *oriC* regions to opposite halves of the cell [80,89,91]. Given the bridging ability of ParB, it is feasible that ParB–ParB interactions may gather the *parS* sites into a single complex. However, microscopy analysis of *C*. *glutamicum* showed that ParB complexed with distinct *parS* sites could be observed as individual subclusters [72]. The presence of a multiple *parS*s adjacent to *oriC* may therefore secure the proper functioning of segregation machinery and improve its robustness.

In *B. subtilis*, *C. crescentus*, *C. glutamicum*, and *S. pneumoniae*, the nucleoprotein ParB–*parS* complexes around *oriC* serve as platforms recruiting SMC–ScpAB condensin complexes and promoting DNA condensation [5,6,71,147,148,149]. Two SMC subunits interact with the kleisin ScpA associated with the dimer of the accessory protein ScpB [150,151,152]. Interactions of ParB–*parS* complexes with SMC complexes direct their binding to DNA in the proximity of *oriC* [5,6,148,152]. Loaded SMC condensin translocates to other parts of the chromosome based on dynamic ATP-dependent transitions between ring-like or open structures [149] and compacts DNA via the loop extrusion mechanism [151,153]. Tethering the two arms of the chromosome together in an ori-ter pattern according to global chromosome organization is dependent on SMC–ScpAB interactions with ParB–*parS* [28,72,148,149,154].

Among bacteria, two other classes of SMC complexes (MukBEF and MksBEF) have also been described. In *E. coli*, MukBEF is required for chromosome segregation but does not facilitate inter-arm contacts, only the long-range co-alignment of chromosomal regions belonging to the same replichores [24,154,155]. No specific loading factor for MukBEF has been identified, suggesting the random loading of condensin complexes on the DNA. In some organisms, such as *P. aeruginosa* or *C*. *glutamicum*, SMC–ScpAB and MksBEF systems co-exist [72,156,157]. Recent study indicated that in *C. glutamicum,* MksBEFG, in contrast to SMC complex, does not contribute to chromosomal DNA-folding or long-range chromosome interactions but instead it seems to be involved in replication control of low-copy number plasmids [72]. In *M. smegmatis* the maintenance of low-copy number plasmids was enhanced by deletion of the *eptC* gene encoding MukB homologue, suggesting important role of EptC in topology of extrachromosomal elements [157]. 

*S. coelicolor*, a representative of Actinobacteria, undergoes drastic changes in chromosome compaction over its complex life cycle. During vegetative growth, elongated hyphal cells are produced with multiple copies of linear uncondensed chromosomes [158]. In sporulating aerial hyphae, unigenomic spores with highly compacted chromosomes are formed. DNA condensation depends on the action of SMC and NAPs specific to sporulation. No indication of ParB–ori complexes recruiting SMC has been reported. Guided by ParA, ParB-bound ori domains were regularly distributed in aerial hyphae before septation in an *smc* mutant but not in the *topA* mutant encoding the single topoisomerase I (TopA) in *S*. *coelicolor* [158]. Topoisomerases are involved in chromosome topology by maintaining adequate DNA supercoiling, for example, to remove the topological tensions arising during transcription, recombination, and chromosome replication [159]. In linear chromosome of *S. coelicolor*, one of TopA’s functions is related to chromosome segregation during sporulation. The depletion of TopA inhibits the efficient separation of paired ParB complexes, which blocks sporulation and retards growth. Sporulation could be at least partially restored by the deletion of *parB*. The direct interactions between ParB–*parS* complexes and TopA suggest that ParB recruits TopA to resolve topological constraints created by ParB interactions with ori domains in *S*. *coelicolor* [158].

## 6. The Role of ParB in the Regulation of Chromosome Replication Initiation

ParB of *B. subtilis* was designated Spo0J, and its partner ParA was designated Soj (**S**uppressor **o**f *spo0**J*** gene), since the first detected phenotype of strain lacking *spo0J* was a sporulation block, whereas the deletion of both genes restored the process [69]. The role of Spo0J in sporulation results from its indirect involvement in the modulation of DnaA transcriptional regulator activity and its replication initiator activity [160] (Table 2). The latter effect is mediated by Soj, which was shown to act as the negative and positive regulator of DnaA, depending on its nucleotide-bound state [161]. Spo0J stimulates ATP hydrolysis by Soj, as well as the dissociation of Soj dimers. Monomeric Soj interacts with DnaA, disturbing its oligomerization, which is indispensable for replication initiation [162].

In the multipartite genomes of *Deinococcus radiodurans* and *V. cholerae* [95,163] the *parB* deletion leads to an increase of the copy number of the cognate replicon. Unlike *B. subtilis*, where Spo0J negatively controls DNA replication through modulation of Soj activity, in these bacteria, direct interactions between ParB and DnaA proteins were detected. *D. radiodurans* ParB1, ParB2, ParB3, encoded by chromosome I, chromosome II, and the megaplasmid, respectively, interact with DnaA and DnaB encoded on chromosome 1 [163]. In the case of *V. cholerae*, both ParA1 and ParB1 directly interact with the DnaA protein [95]. The molecular mechanisms and significance of these interactions remain unclear.

## 7. Involvement of ParB in The Nucleoid Occlusion

Deletion of the *spo0J* gene from the *B. subtilis* chromosome results not only in aberrant DNA replication and sporulation but also affects cell division [70]. Elongated cells were detected in populations of strains deprived of Spo0J or both, Spo0J and Soj, but not in the strain lacking only Soj [70]. In *B. subtilis,* two systems controlling cell division were described (for a review, see [185]). The Min system hampers the formation of the cytokinetic Z-ring composed of tubulin like FtsZ protein close to the cell poles [186]. The nucleoid occlusion (NO) system prevents premature Z-ring formation over the nucleoid until most of the chromosomal DNA has been segregated. Spo0J together with its paralog, Noc [187,188,189], block premature Z-ring assembly, preventing chromosome guillotining in the dividing cells. The deletion of either *noc* (initially named *yyaA*) or *spo0J* results in aberrant cell division, and the deletion of both *noc* and *spo0J* has a synergistic effect, potentiating the aberrations [190].

## 8. The Interactions of ParB with Topological Determinants during Cell Division 

Another aspect of ParB’s involvement in the cell division process is its interplay with proteins located at the cell poles, such as DivIVA in *B*. *subtilis* [164] (Table 2). DivIVA is a highly conserved component of the Min system (equivalent to the *E. coli* MinE protein in the MinCDE system) in a wide range of Gram-positive bacteria (for a review, see [191]). The biological functions and significance of DivIVA vary among different bacterial species. However, the primary role of DivIVA (as one of the Min proteins) seems to be the proper positioning of the cell division site. Since Spo0J–DivIVA interactions are observed in *B. subtilis* only during the early stages of sporulation when DivIVA supports Spo0J-*parS* guided orientation of the chromosome and its polar attachment, it is postulated that DivIVA is also involved in the molecular switch between vegetative cell division and spore formation [164]. 

Interactions between DivIVA and ParB were also detected in the non-sporulating bacteria *S. pneumoniae* [169] and *D. radiodurans* [180,181]. In the latter, DivIVA interacts with various ParBs encoded by the multipartite genome, with ParB1 (chrI), ParB2 (chrII), and ParB3 and ParB4 from the megaplasmid and plasmid, respectively. In the case of *D. radiodurans*, ParB1, ParB3, and ParB4 also interact with another Min protein—MinC that functions as an FtsZ polymerization inhibitor [180]. 

It should be noted that, in some cases, ParB may be indirectly engaged in ori domain anchoring (Table 2). In *Mycobacterium smegmatis*, ParA, instead of ParB, interacts directly with DivIVA [166]. In *S. coelicolor*, both ParA and ParB are involved in localization of the segrosome in hyphae [171]. However, only ParA interacts with so-called ‘polarisome’ or tip-organizing complex (TIPOC), which includes DivIVA and the coiled-coil protein, Scy, and anchors the segrosome at the tips. Whereas ParA and Scy direct interactions have been confirmed [173], there is no evidence for ParA (or ParB) and DivIVA interactions in *S. coelicolor* [191]. In *C. crescentus*, ParA mediates the ParB–DNA complex’s positioning at the new cell pole via interactions with the TipN protein [178]. In *V*. *cholerae,* ParB1–*oriC1* complex is targeted to the cell pole via interactions between the polar protein HubP and ParA1 [182].

In α-proteobacteria, lacking Min and NO systems, ParB interacts with PopZ, polar organizing protein Z (DivIVA functional equivalent), and MipZ, the mid-cell positioning of the FtsZ protein [175,177,192]. PopZ is a key component in the regulation and coordination of chromosome replication and partitioning in *C. crescentus*, a bacterium with a dimorphic lifestyle (for a review, see [193]). In this organism, cell division leads to the formation of a mobile swarmer cell and a non-mobile stalker cell. However, only the latter is able to duplicate [194]. The ori domain of the longitudinally oriented chromosome is anchored to the stalked pole by direct interactions between ParB–*parS* and PopZ [175]. Immediately after DNA replication initiation, one of the duplicated ori domains begins translocation towards the opposite pole, assisted by the ParAB-*parS* system. Simultaneously, the mono-polar localization of PopZ is switched to the bi-polar localization, and the transferred ParB–*parS* complex (ori domain) is attached to the cell membrane via the PopZ protein complex at the swarmer pole [192]. At this stage of the chromosome segregation also ParA directly interacts with PopZ protein [176].

In the marine alpha-proteobacterium, *Hyphomonas neptunium*, which proliferates by bud formation at the tip of a stalk-like cellular compartment, a unique two-step chromosome segregation process occurs [83]. Initially, two newly replicated ori domains are segregated to opposite poles of the mother cell via a ParAB-*parS* dependent mechanism. When the bulk of the chromosome has been replicated, the cell produces a bud (swarmer cell), and a next segregation step largely independent of replication begins. This step involves the translocation of a stalk-proximal ParB–origin region through the stalk to the bud compartment by an unknown mechanism. PopZ’s homolog from *H. neptunium* associates with the ParB–*parS* complex in swarmer cells and in the bud compartment at a later stage of the cell cycle, possibly acting as a tether for the ori domain at the new bud pole [83].

DNA segregation and the PopZ mediated attachment of ParB–*parS* complexes at the cell poles are critical for the appropriate positioning and formation of the cytokinetic Z-ring. In *C. crescentus*, ParB is also involved in the formation of a bi-polar gradient of MipZ, a polymerization inhibitor of the FtsZ protein, and both ParB and MipZ are indispensable [177]. ParB stimulates the formation of MipZ dimers, negatively affecting the polymerization of FtsZ and hence Z-ring formation [177]. MipZ dimers are also able to bind DNA in a non-specific manner [195]. This binding stimulates MipZ ATPase activity and promotes dimer dissociation, which results in a release of MipZ monomers from the DNA [196]. In *C. crescentus*, MipZ forms a bipolar comet-like gradient with the highest concentration of MipZ in the cell poles and the lowest concentration in the midcell before division. This distribution of MipZ depends on the ParB–*parS* complex movement from the stalked pole to the swarmer pole and promotes Z-ring positioning at the midcell. In another representative of α-proteobacteria, *Rhodobacter sphaeroides*, the bipolar comet-like gradient of MipZ was not detected [183]. Detailed studies revealed that *R. sphaeroides* MipZ, similarly to *C. crescentus* MipZ, interacts with ParB as a monomer and inhibits FtsZ as a dimer. However, in this organism, MipZ dimers are never present at the cell poles but form a ring-like structure at the midcell, facilitating Z-ring assembly and stability rather than Z-ring positioning (as was postulated for *C. crescentus*) [183]. 

Finally, a functional analysis of two MipZ-like proteins encoded by *mipZ1* and *mipZ2* in *Magnetospirillum gryphiswaldense* revealed that MipZ1 is crucial for proper cell division, and MipZ2 has only a minor effect on this process [184]. MipZ1 interacts with ParB and forms a bipolar comet-like gradient akin to MipZ of *C. crescentus*, while MipZ2 localizes to the cell division site similar to the MipZ of *R. sphaeroides* [184].

The role of ParB in cell division was also postulated in *C*. *glutamicum* [73]. Similarly to *C. crescentus*, *C. glutamicum* lacks Min and NO systems. *C. glutamicum* ParB interacts not only with ParA but also with the ParA homologue, the PldP protein (with a 62% sequence similarity). Analysis of the *pldP* mutant showed significant defects in the cell division, comparable to *E. coli* and *B. subtilis* mutants in *min* genes [73]. *In vitro*, ParB interacts not only with PldP but also with the FtsZ protein. It is not clear at which stage of the cell cycle the crosstalk between DNA segregation and cell division takes place. A fluorescently labelled PldP protein can be detected as visible foci near the cell division site, but diffused PldP is also present in the cytoplasm [73].

## 9. ParB’s Role in Capsule Formation in *S. pneumoniae*

In *S*. *pneumoniae*, ParB interacts with DivIVA during cell division. It has also been shown that ParB participates in specific crosstalk between DNA segregation, cell division, and capsule formation in this organism [168,170]. *S*. *pneumoniae’s* incomplete Par(A)B-*parS* system consists of four *parS* sites close to *oriC,* as well as an orphan *parB* gene [197]. The deletion of *parB* impairs chromosome segregation but only in a minor way and has no influence on bacterial growth [71]. Recent reports have shed light on the role of *S*. *pneumoniae* ParB, showing that it cooperates with two proteins, CpsD and RocS [168,170]. Capsular polysaccharide protein D (CpsD), a ParA-like ATPase, is a component of a protein complex involved in the synthesis and export of capsular polysaccharide. Together with the membrane associated CpsC, it forms a tyrosine autokinase. Autophosphorylated CpsD stimulates ParB–*parS* binding and promotes chromosome segregation, as well as cell division and capsule formation. Non phosphorylated CpsD delays ParB–*parS* translocation to the cell equator and negatively affects cell division [168]. Another ParB partner, RocS (Regulator of chromosome Segregation), was initially identified as a CpsD partner in the control of cell division [170]. RocS is a non-specific DNA binding protein, with a N-terminal MarR-like HTH motif and a C-terminal MinD-like cell membrane binding helix [170]. RocS and ParB seem to have partially overlapping functions in *S. pneumoniae,* and a double deletion of *rocS* and *parB* is lethal. However, the lack of RocS has a more deleterious effect on DNA segregation than ParB deficiency alone [170]. In contrast to ParB, RocS is not involved in capsule formation but is directly involved in cell division control through CpsD. Thus, in *S. pneumoniae*, this unique system, involving ParB, RocS, and CpsD (CpsC) proteins, coordinates and secures DNA segregation, cell division, but also capsule formation, which is critical for bacterial virulence [170]. 

## 10. Impact of ParBs on Gene Expression

The autoregulation of partition operon expression is a common feature of plasmidic systems [41]. However, in the Class Ia type of *par* loci, ParA acts as a repressor, binding in the promoter region of the *parAB* operon, whereas ParB usually works as a co-repressor [41,99,100]. The known examples of plasmidic ParBs of Class Ia playing the role of the global transcriptional regulators include the KorBs of IncP-1 plasmids [126,198,199] and related KorB of IncU plasmids [200].

**Figure 4:**
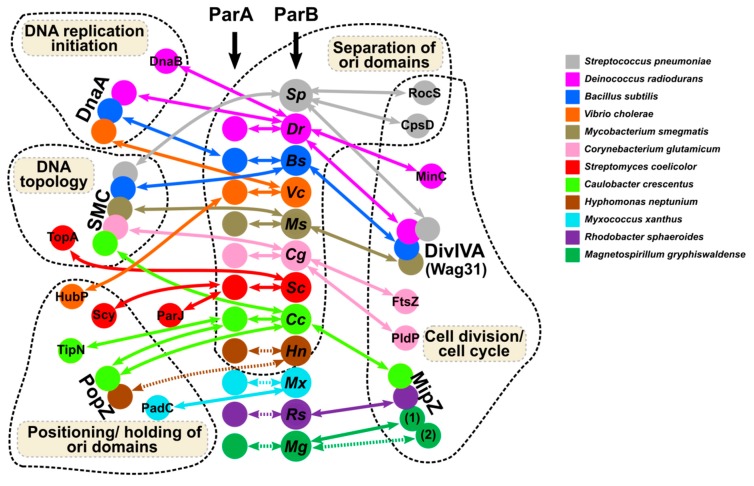
Network of ParBs interactions with ParAs and other partners coordinating chromosome segregation with various cellular functions in the analyzed bacterial species. The dashed arrows indicate interactions not yet confirmed experimentally.

For chromosomal ParB proteins, artificial insertion of the *parS* sequence upstream of the *repA* promoter in the test plasmid was used to demonstrate the negative effect of *P. aeruginosa* ParB binding to *parS* on expression of the adjacent genes [64]. The flexibility of ParBs in self-interactions via the use of NTDs and CTDs, specific and unspecific interactions with DNA and interactions with multiple partners (Figure 4) may facilitate the formation of the expanded ParBs network, potentially influencing gene expression. 

In recent years, the relation between ParB binding to chromosomal DNA and the expression of loci close to the corresponding *parS* sites has been systematically investigated using a combination of ChIP methods and genome-wide transcriptomic approaches. In *B*. *subtilis*, Spo0J was observed to bind to and around 10 *parS* sequences. However, a lack of Spo0J did not affect the expression of genes adjacent to these sites [68]. Spo0J deficiency resulted in an elevated expression of only the *fruR* gene (encoding a putative transcriptional regulator), whereas the mRNA levels of nine other genes, among them, the sporulation genes *spoIIGA, cotE, cotG, spoIIAA,* and *sigE,* were decreased [68]. This negative effect of sporulation gene regulation can be explained by the influence of Spo0J deficiency on the Soj mediated activation of the Sda replication check point [160].

The influence of ParB on gene expression was also studied in *V*. *cholerae*, a bacterium with two chromosomes, each encoding their own partitioning system (*parABS1 and parABS2)*. Chromatin precipitation combined with the microarray technique (ChIP-chip) confirmed that ParB1 bound to three *parS* motifs and spread for 16 kbp [132]. The transcriptomic analysis of the Δ*parB1* mutant revealed a changed expression of only three genes in the region occupied by ParB and several more outside of this region. Cloning promoters of these three genes upstream of the promoter-less *lacZ* on the plasmid eliminated the regulatory effect of ParB1, indicating that genetic localization and/or DNA topology play a role in the regulation of these promoters by ParB1. The role of ParB1 in controlling gene expression outside of the highly ParB-enriched zone was postulated to be indirect through interactions with other regulatory proteins [132].

A similar transcriptional analysis in *S. pneumoniae* showed that a lack of ParB affected the expression of eight genes. However, none of these genes were located in proximity to the four *parS* sequences identified in this organism [130]. Significantly, ParB deficiency resulted in a modest increase in the expression of the *comCDE* operon involved in the regulation of competence and located 5 kbp away from *parS* (−1.6°) [130]. Mutating *parS* (−1.6°) to block ParB binding was sufficient to induce *comCDE* expression, suggesting that a ParB complex formed at a single *parS* may influence the expression of promoters at a distance. Attempts to recapitulate this effect with the insertion of a strong synthetic promoter in the region failed, suggesting a highly selective mechanism by which the ParB complex at *parS* (−1.6°) influences *comCDE* expression and, as a result, the development of the competence cascade [130]. 

The genome wide transcriptome analyses presented above indicate that ParB deficiency results in minor changes in the cell transcriptomes of the tested species. However, the analysis of ParB deficient *P*. *aeruginosa* cells from exponentially growing cultures in rich medium showed expression changes for 1166 genes, which is around 20% of all loci [201]. Additionally, global changes in the transcriptome were also observed in ParA deficient cells; 697 genes showed altered expression in the *ΔparA* mutant, and 77% of them overlapped with the gene pool affected by ParB deficiency [201]. This overlap is not surprising, as previous data indicated that ParA deficiency in this organism promotes ParB degradation [92]. The wide range of changes in the transcriptomes of *par* mutants reflect a wide spectrum of various growth defects. The lack of *parA* or *parB* in *P. aeruginosa* leads up to a 1000-fold increase in the number of cells with defects in their chromosome segregation, extended division times, longer cells, altered colony morphology, and impaired swimming and swarming motility [92,202]. We cannot exclude that observed changes in the expression of some genes result from the aberrations in ParB and/or ParA dependent processes like DNA segregation although anucleate cells constitute only 2%–4% of population under analyzed conditions. Significantly, not only depletion, but also mild overproduction of ParB protein lead to global transcriptome changes in *P. aeruginosa* [203].

*P. aeruginosa* ParB binds to a cluster of four *parS* sequences (*parS*1-4) in the vicinity of *oriC* to form a large nucleoprotein complex, and the binding to one of these sites is required and sufficient for proper chromosome segregation [89,90,91]. The analysis of the mRNA levels of genes adjacent to *parS*1-4 showed that direct ParB interactions with intergenic *parS*3 and *parS*4 motifs located upstream of *PA0011* and *PA0013*, respectively, repressed their expression, while the expression of other genes in the analysed region was unchanged in the *parB* and *parS*1-4 deficient cells [203]. Concomitantly, a mild overproduction of ParB led to a more pronounced transcriptional silencing of the majority of genes in the vicinity of the *parS*1-4 cluster. This observation suggests that the size, composition, or stability of the ParB nucleoprotein complex may dictate the effect on gene expression [203]. Strikingly, our recent study demonstrated that in *P. aeruginosa,* ParB was bound to numerous sites containing a heptanucleotide half-*parS*. However, the binding to half-*parS* sites may not directly account for the transcriptome changes ParB exerts. Only 15% of ParB enriched half-*parS* sites localizes in intergenic regions with putative transcriptional initiation signals [90]. Binding of *P. aeruginosa* ParB to hundreds of specific DNA motifs may have a great impact on chromosome topology (Figure 3c). ParBs from several other bacterial species also bind to GTTCCAC and GTTTCAC sequences (Figure 3b), indicating that *P. aeruginosa* ParB is not unique in this mode of interaction. The significance of these interactions in cell processes (including gene expression regulation) requires further studies.

## 11. Conclusions and Future Perspectives

ParAB-*parS* systems are encoded close to *oriC*s in the chromosomes of most bacterial species [41,59]. The primary role of ParAB-*parS* systems is undoubtedly the segregation of newly replicated DNA. ParB binding to *parS*s in the proximity of *oriC*, structuring ori domains, directing their motion with the help of the ParA partner, and the anchoring of ori domains at the defined locations are vital for accurate segregation of the genomes. With a few exceptions (Table 1), this system is not essential for cell survival but rather an accessory to hypothetical but attractively more universal entropy-driven DNA segregation machinery [204,205]. In some species, like *T*. *thermophilus*, the ParAB-*parS* system seems not to be involved at all in DNA segregation [98]. New sophisticated molecular biology methods revealed the details of chromosome segregation, providing insight into the formation and structure of the ParB nucleoprotein complex, as well as the mechanisms responsible for its relocation to specific cell positions. Nevertheless, the picture of this process is far from complete. The diversity of the Par partners (Table 2 and Figure 4) and the species-specific requirements for cell cycle proceedings still lead to new exciting discoveries [83,206]. 

ParB proteins seem to be engaged in crosstalk between DNA segregation, DNA replication, cell growth, and division. The synchrony of these processes in non-compartmentalized cells emphasizes the significance of such crosstalk (reviewed in [193]). The ParBs domain’s composition and structural flexibility facilitates various modes of interactions with specific (*parS* and half-*parS* sites) and non-specific DNA and points to multiple interfaces for self-interactions and cooperation with different protein partners. The involvement of ParBs in a variety of cell functions directly through direct interactions with vital cell components or indirectly using their primary partners, ParAs, is summarized in Figure 4. To achieve that, they target similar main players in different species (DnaA, SMC, DivIVA, MipZ) or adapt to species-specific factors playing the same function, such as a polar positioning. 

The role of ParB as a transcriptional regulator has been shown in a limited number of species and for a limited number of genes, not necessarily located close to the *parS* sites. The most intriguing is the biological significance of ParB binding to half-*parS*s observed in some species, despite the common occurrence of these motifs in the genomes. In *P. aeruginosa* and a few other species ParB binds to hundreds of half-*parS* sites (Figure 3b). These interactions may induce topological constraints leading to the observed ParB role as a global transcriptional regulator in *P. aeruginosa* [201]. 

One of the most poorly understood aspects of ParBs functioning is the nature of molecular switches that control the level and activity/conformation of ParBs. Par proteins are not as abundant as histone-like proteins [68,92]. The level of Par proteins, possibly influencing their activities, could be controlled by different mechanisms. The regulation of *parB* and *parA* expression has only been partially studied in a limited number of bacteria species (e.g., [84,132,207,208]). Alternatively, the levels of Par proteins could be modulated by cell proteases, e.g., depending on the phase of growth [92].

The observation that ParB loading at *parS* and sliding on DNA requires CTP binding [122,140,141], a key metabolite in DNA and phospholipids synthesis, adds CTP to the players possibly regulating the ParB–DNA interactions. The motif containing amino acid residues responsible for CTP binding and ParB CTPase activity is one of the most conserved elements within the ParB protein sequences [122,141]. This suggests that CTP binding and hydrolysis may be crucial for proper ParB functioning, not only in the abovementioned *B. subtilis* [122]*, M. xanthus* [141] and *C. crescentus* [133], but also in other bacteria encoding ParAB-*parS* systems.

An increasing number of important bacterial proteins have been shown to undergo post-translational modifications (PTMs), such as phosphorylation or acetylation [209,210,211]. *M. tuberculosis* ParB is phosphorylated by Serine/Threonine Protein Kinases (STPKs), including PknB, a key component of the signal transduction pathway that regulates, for example, cell division and the survival of the pathogen in the host [212,213]. This PTM negatively affects ParB’s DNA binding properties in vitro, as well as ParB interactions with the cognate ParA protein partner. The regulation of ParB activity by direct phosphorylation or acetylation is not the only way to affect its activity, as its partners’ activity may be dependent on PTM. For example, the functions of *S. pneumoniae* ParB depend on interactions with the membrane bound CpsC/D tyrosine auto-kinase [168]. We also cannot exclude the possibility that CpsC/D phosphorylates *S. pneumoniae* ParB, like in the case of *M. tuberculosis* auto-kinases from the STPK family [212]. 

The biological roles of chromosomally encoded ParBs and the molecular basis of their functions are more complex than was previously thought. It is clear that despite the significant conservation of chromosomal ParAB-*parS* systems, these systems evolved independently in different bacterial species. ParBs seem to be excellent models for studying many aspects of bacterial molecular biology and genome evolution. 

## Figures and Tables

**Table 1:** Characterization of chromosomally encoded *par* systems.

Species	*parS* Sites Total/in ori Domain/Consensus	*par* Genes	Anucleate Cells in *parB* Mutant	Other Phenotypes associated with Mutation in *parB* Gene
**Gram-positive:**				
Firmicutes				
*Bacillus subtilis*	10/**8**/TGTTNCACGTGAAACA [68]	non-essential	1%–2% (wt 0.02%) [69]	Defect in sporulation, elongated cells, increased amount of DNA *per* cell, overreplication: >2 foci corresponding to *ori* in 35.4% cells (wt 15.3%), disturbed replication control and *ori* separation [70], impaired SMC loading (lack of foci) [6]
*Streptococcus pneumoniae*	4/**4**/AGTTTCACGTGAAACT [71]	non-essential, no *parA*	0.8% at 30 °C to 3.5% at 37 °C (wt 0%) [71]	No apparent growth defects, mild perturbations in chromosome segregation, decreased SMC loading near origin [71]
Actinobacteria				
*Corynebacterium glutamicum*	10/**10**/TGTTNCACGTGAAACA [72]	non-essential	43.8% in MMI medium, 11.6% in LB (wt 0%) [73]	Reduced growth rate in MMI medium, growth not affected in an LB medium, altered cell morphology (almost “coccoid” cells and elongated anucleate cells) [73], impaired SMC loading (lack of visible foci) [72]
*Mycobacterium smegmatis*	3/**3**/GTTTCACGTGAAAC [74]	non-essential	10.3% (wt 0.8%) [74]	Elongated cells, overreplication, disturbed septa formation, origin positioning, and chromosomal topology [75]
*Streptomyces coelicolor*	21/**21**/tGTTTCACCTGAAACa [76]	non-essential	13%–17.4% anucleate spores (wt 1%–2%) [77,78]	Disturbed sporulation, reduced growth rate, elongated cells, premature and irregular Z-ring formation [79]
**Gram-negative:**				
Alphaproteobacteria				
*Caulobacter crescentus*	5/**5**/t/cGTTt/cCACGTGAAAca [80,81]	essential		Indispensable, severe chromosome segregation defects, ParB depletion results in defective Z-ring formation and cell division, formation of long polyploid cells [82]
*Hyphomonas neptunium*	2/**2**/TGTTTCACGTGAAACA [83]	essential	anucleate buds [83]	*parB* mutants could not be obtained, depletion of ParA blocks cell division [83]
Betaproteobacteria				
*Burkholderia cenocepacia*	chrI: 2/**2**/tGTTNCACGTGAAACa chrII: 6/**6**/gTTTATGCGCATAAAc [84,85,86,87]	non-essential	1–14% (depending on mutated system) [85]	Reduced growth rate, reduction in cell size, compromised viability, defects in ori positioning [85]
Deltaproteobacteria				
*Myxococcus xanthus*	22/**22**/TGTTCCACGTGGAACG [88]	essential	ParB depletion: 1% after 24 h, 10.1–21.6% after 36–48 h [88]	ParB depletion: aberrant cell morphology, anomalies in DNA segregation and cell death [88]
Gammaproteobacteria				
*Pseudomonas aeruginosa*	9/**4**/TGTTCCACGtGGAACa half-*parS*s GTTCCAC or GTTTCAC [89,90,91]	non-essential	2–4% in LB medium, to 7% in an M9 medium (wt < 0.01%) [92]	Reduced growth rate, 10–15% increase in cell size and 10% longer generation time, altered colony morphology, affected motility; decreased ParA stability [92]
*Pseudomonas putida*	?*/**3** TGTTCCACGTGGAACA [63]	non-essential	5–10% in minimal medium during the transition from exponential to stationary phase [93]	Defects in chromosome partitioning, abnormal cell morphologies during the deceleration phase of growth independent of the medium used [63,93]
*Vibrio cholerae*	chrI: 3/**3**/NGTTNCACGTGAAACN chrII: 10/**9**/NTTTACANTGTAAAN [94]	non-essential chr1 essential chr2	no change in *parB1* mutant [95]	Increased frequency of replication initiation, disturbed ori positioning in cell poles [95], no segregation defect for *V. cholerae* chrI [94]
Deinococci				
*Deinococcus radiodurans*	chrI: 3/**1**/NGTTTcgcGtgaAACN [96]	non-essential	8%—13% for ∆*parB1*, (wt >1%) [96]	Reduced growth rate for ∆*parB1* [96]
*Thermus thermophilus*	1/**1**/TGTTTCCCGTGAAACA [97]	non-essential	3% for ∆*parAB* (wt 1.2%) [97]	No apparent growth defects for ∆*parAB* [97,98]

**Abbreviations: chrI/chrII**- primary/secondary chromosome in the multipartite genome; **wt**—wild-type; ?* - only contig with *P. putida oriC* was analyzed for presence of *parS*s in the cited reference.

**Table 2:** Interactions of ParBs (or ParB–*parS* complexes) with protein partners and the methods used for their analysis.

Species	ParBs (or ParB–*parS* complexes) Protein Partners			
	ParA dependent re-locations of ParB–*parS* complexes	Chromosome *ori* domain modelling	Localization/anchoring Cell division control	Replication initiation regulation
***Bacillus subtilis***	+ [70]	**SMC**; Interplay between SMC and ParB–*parS* complexes shown by ChIP-seq, FM, and mutational analysis (deletion of *parB* or *parS* sites affect SMC foci formation) [5,6]	**DivIVA**; Direct interaction confirmed by Co-IP, Spo0J may participate in switching between vegetative growth and sporulation [164]	**#DnaA**; Spo0J induces NTPase activity of Soj (ParA), the DnaA regulator [160,161]
***Myxococcus xanthus***	ND		**#PadC**; PadC mediates the binding of ParA to BacNOP cytoskeletal proteins (BLI); ParB may interact with BacNOP through a yet unidentified protein (pull-down) [141,165]	
***Mycobacterium smegmatis***	+ [166]	**SMC**; Interplay between SMC and ParB–*parS* complexes shown by FM and mutational analysis (deletion of *parB* affects SMC foci formation) [75]	**#DivIVA (Wag31)**; ParA interacts with DivIVA and mediates nucleoid anchoring at the cell poles, co-localisation confirmed by the FM of labelled proteins, interactions proven by BACTH and pull-down [166,167]	
***Streptococcus pneumoniae***	lack of ParA; **CpsD**, Walker-type ATPase, is involved in ParB–*parS* movements [168]	**SMC**; Interplay between SMC and ParB–*parS* complexes shown by FM and mutational analysis (deletion of *parB* affects SMC foci formation) [71]	**DivIVA**; Direct interactions confirmed by Co-IP and BACTH [169] **RocS**; Co-localisation confirmed by FM of labelled proteins, direct interactions confirmed by Co-IP and MST [170], RocS and ParB participate in DNA segregation; CpsD*; Co-localisation confirmed by FM of fusion proteins, direct interaction (CpsD phosphorylation dependent) confirmed by Co-IP and MST, CpsD and ParB cooperate in coordination with DNA segregation, cell division, and capsule formation [168]	
***Streptomyces coelicolor***	+ [171]	**TopA**; Interactions with ParB–*parS* complexes proven by pull-down, Co-IP, ChIP-seq; these interactions may support chromosome resolution [158]	**#ParJ**; ParJ negatively regulates the ParA polymerization indispensable for chromosome segregation, direct interaction confirmed by BACTH and SPR [172] **#Scy**; The polarity determinant interacts with ParA, co-localisation confirmed by FM of labelled proteins, direct interactions confirmed by BACTH, co-purification and SPR, ParA and Scy coordinate growth and chromosome segregation [173]	
***Caulobacter crescentus***	+ [174]	**SMC**; Interplay with ParB–*parS* complexes shown by Hi-C, ChIP-seq, and mutational analysis, ParB dependent loading also detected on differently positioned *parS* sites [151]	PopZ*; Co-localisation confirmed by the FM of labelled proteins, direct interactions confirmed by Co-IP and SPR, EMSA (PopZ with ParB–*parS* complexes), PopZ–ParB–*parS* complexes mediate chromosome anchoring to the cell poles [175] #PopZ*; PopZ interacts also with ParA, co-localisation confirmed by FM of labelled proteins, direct interaction confirmed by Co-IP and SPR, interactions between PopZ and ParA mediate chromosome movement towards the swarmer pole [176] MipZ*; Co-localisation confirmed by the FM of labelled proteins, direct interactions confirmed by SPR and EMSA (MipZ with ParB–*parS* complexes), ParB–MipZ complexes control Z-ring positioning [177] **#TipN**; The polarity determinant interacts with ParA, direct interactions proven by FM and SPR, ParA–TipN complexes anchor ParB–*parS* (*ori* domain) to the new cell pole [103,178]	
***Corynebacterium glutamicum***	+ [73]	**SMC**; Interplay with ParB–*parS* complexes shown by FM, ChIP-seq, deletion of *parB* or *parS*s results in the loss of SMC loading and foci formation [72]	**FtsZ**; Direct interactions confirmed by BACTH [73] PldP*; Direct interactions confirmed by BACTH [73], putative new cell division control system	
***Deinococcus radiodurans***	+ [179]		**MinC**; Interactions with ParB1 (chrI), ParB3 (megaplasmid), and ParB4 (plasmid) confirmed by BACTH [180]; **DivIVA**; Interactions with ParB1, ParB2, ParB3, and ParB4 confirmed by BACTH and Co-IP [181]; ParB1 interacting with DivIVA may mediate chromosome anchoring to the cell poles and through interactions with FtsZ inhibitor MinC may affect the cell division. Interactions of DivIVA and MinC with ParB2-4 coordinate the segregation of multipartite genome and cell division.	**DnaA**, **DnaB**; interactions with ParB1(chrI) ParB2 (chrII) and ParB3 (megaplasmid), confirmed by BACTH and Co-IP; these interactions probably regulate DNA replication initiation [163]
***Vibrio cholerae***	+ [107]		**#HubP**; ParA1 interacts with HubP to mediate nucleoid anchoring at the cell poles; interactions confirmed by the FM of fusion proteins and BACTH [182]	**DnaA**; Interactions with ParB1 confirmed by BACTH, a proposed role in the regulation of replication initiation [95]
***Hyphomonas neptunium***	+ [83]		PopZ*; Co-localisation with ParB–*parS* in the swarmer cells and in the bud compartments confirmed by the FM of tagged proteins [83]	
***Rhodobacter sphaeroides***	ND		MipZ*; Co-localization demonstrated by the FM of fusion proteins; direct interactions confirmed by BACTH; the ParB–MipZ system supports the Z-ring assembly and its stability [183]	
***Magnetospirillum gryphiswaldense***	ND		MipZ1*; Co-localization demonstrated by the FM of fusion proteins; direct interactions confirmed by BLI, ParB–MipZ1 system controls Z-ring positioning; MipZ2* may also interact with ParB [184]	

**ND**: not determined; **#:** Indirect interactions mediated by the cognate ParAs; *: ParA-like protein, Walker-type ATPase; **FM**: Fluorescence microscopy; **BACTH**: Bacterial two hybrid system; **Co-IP**: Co-immunoprecipitation; **Hi-C:** chromosome conformation capture; **ChIP-seq**: Chromatin immunoprecipitation followed by DNA sequencing; **SPR**: Surface plasmon resonance; **EMSA:** Electrophoretic mobility shift assay; **MST**: Microscale thermophoresis; **BLI**: Bio-layer interferometry.
